# Construction of Pará rubber tree genome and multi-transcriptome database accelerates rubber researches

**DOI:** 10.1186/s12864-017-4333-y

**Published:** 2018-01-19

**Authors:** Yuko Makita, Mika Kawashima, Nyok Sean Lau, Ahmad Sofiman Othman, Minami Matsui

**Affiliations:** 1 Synthetic Genomics Research Group, Biomass Engineering Research Division, RIKEN Center for Sustainable Resource Science (CSRS), 1-7-22 Suehiro-cho, Tsurumi-ku, Yokohama, Kanagawa 230-0045 Japan; 2 Centre for Chemical Biology, Universiti Sains Malaysia, 11900 Bayan Lepas, Penang, Malaysia; 3 School of Biological Sciences, Universiti Sains Malaysia, 11800 Minden, Penang, Malaysia

**Keywords:** *Hevea brasiliensis*, Transcriptome, Gene annotation, Database, Latex biosynthesis, *cis*-1,4-polyisoprene, R-gene

## Abstract

**Background:**

Natural rubber is an economically important material. Currently the Pará rubber tree, *Hevea brasiliensis* is the main commercial source. Little is known about rubber biosynthesis at the molecular level. Next-generation sequencing (NGS) technologies brought draft genomes of three rubber cultivars and a variety of RNA sequencing (RNA-seq) data. However, no current genome or transcriptome databases (DB) are organized by gene.

**Results:**

A gene-oriented database is a valuable support for rubber research. Based on our original draft genome sequence of *H. brasiliensis* RRIM600, we constructed a rubber tree genome and transcriptome DB. Our DB provides genome information including gene functional annotations and multi-transcriptome data of RNA-seq, full-length cDNAs including PacBio Isoform sequencing (Iso-Seq), ESTs and genome wide transcription start sites (TSSs) derived from CAGE technology. Using our original and publically available RNA-seq data, we calculated co-expressed genes for identifying functionally related gene sets and/or genes regulated by the same transcription factor (TF). Users can access multi-transcriptome data through both a gene-oriented web page and a genome browser. For the gene searching system, we provide keyword search, sequence homology search and gene expression search; users can also select their expression threshold easily.

**Conclusion:**

The rubber genome and transcriptome DB provides rubber tree genome sequence and multi-transcriptomics data. This DB is useful for comprehensive understanding of the rubber transcriptome. This will assist both industrial and academic researchers for rubber and economically important close relatives such as *R. communis*, *M. esculenta* and *J. curcas*.

The Rubber Transcriptome DB release 2017.03 is accessible at http://matsui-lab.riken.jp/rubber/.

**Electronic supplementary material:**

The online version of this article (10.1186/s12864-017-4333-y) contains supplementary material, which is available to authorized users.

## Background

Natural rubber is an indispensable material for many industrial applications such as in tires and medical devices [1]. Although more than 2500 plants produce latex, currently the Pará rubber tree (*Hevea brasiliensis* Muell. Arg.) is the only main commercial source for rubber production [2]. Even compared with petro-chemically synthesized rubber, natural rubber has advantages in adhesion, elasticity and durability.

Natural rubber is produced from specialized differentiated cells called laticifer cells in the outer layer of bark. Rubber latex is composed of rubber serum and rubber particles where *cis*-1,4-polyisoprene biosynthesis occurs. The molecular mechanism of rubber production is not well understood. Additionally, disease-resistance is an important trait to identify for rubber research and breeding. Rubber trees are susceptible to several fungal infections including South American leaf blight (*Microcyclus ulei*) and different cultivars show different sensitivity [3].

To accelerate molecular biological research in *H. brasiliensis*, we first determined its draft genome sequence and annotated 84,443 protein-coding genes [4]. After the rubber tree genome was determined, transcriptome data is important to identify gene expression level and precise gene structure, such as transcription start sites (TSS) and isoforms. Currently, RNA-seq is the most widely used transcriptome technology, providing gene expression levels in many conditions and tissues. For the rubber tree, latex where *cis*-1,4-polyisoprene biosynthesis occurs is the key tissue to understand the mechanism of rubber production. Many researchers determined gene expression of latex in different cultivars or conditions [5–9]. Although RNA-seq is a powerful tool to know gene expression, it is difficult to predict full-length splice isoforms and TSSs accurately. For better understanding of transcription in rubber tree, we constructed full-length cDNA libraries and determined their sequence with Sanger and Illumina [10]. Pootakham et al. released Pacific Biosciences (PacBio) Isoform sequencing (Iso-Seq), single-molecule real-time long-read isoform sequencing in BPM24 cultivar [11].

When we predict 5′-end with EST and/or RNA-seq, we tend to predict the longest TSSs instead of major expressed TSSs [12]. To know major expressed TSS in different tissues, we applied cap analysis gene expression (CAGE) method that captures the 5′ end of the transcribed and capped mRNAs. CAGE provided us with a genome wide single base-pair resolution map of TSSs [13].

One of the main difficulties for gene annotation in non-model plants is the number of genes with unknown functions. In our case, 22,991 genes are rubber specific and functionally unknown and 33,213 genes have homologous sequences but are still functionally unknown. To overcome the problem, we carried out co-expressed analysis to predict functionally related gene groups. We can expect genes regulated by the same transcription factor (TF) or genes involved in the same biological pathway to show a similar expression pattern. There are many co-expression databases in plant [12, 14]. In this DB, we show the top 20 similarly expressed genes in rubber.

## Construction, content and utility

### General structure and content of rubber Transcriptome DB

**Fig. 1:**
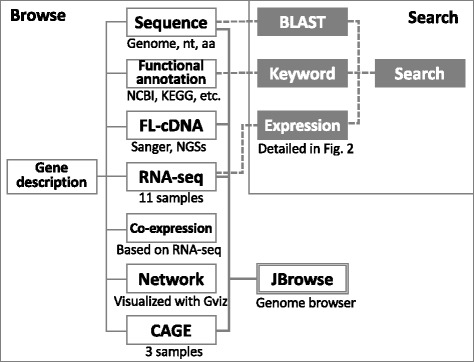
The structure of rubber transcriptome database. Gene-oriented DB has functional annotation and multi-transcriptome data. Based on 11 RNA-seq data, we calculated co-expressed genes and their networks. All transcriptome data are available from JBrowse, a fast visualization genome browser. Three types of search systems are also available. See Fig. 2 for the details of the gene expression search

Based on our original draft genome sequence of *H. barasiliensis* RRIM 600, we predicted 84,443 protein-coding genes [4]. Users can access functional annotation and three kinds of transcriptome data through web pages organized by gene. The basic structure of our DB is summarized in Fig. 1. Users can access functional annotation and links to original databases; NCBI protein, KEGG, UniProt (both Swiss-Prot and TrEMBLE) and Gene Ontology [15–18]. We also provide multi-transcriptome data of our original full length cDNA (FL-cDNA), RNA-seq and CAGE, and publicly available ESTs, RNA-seq and Iso-Seq (Table 1) [4, 10, 11, 19–22]. Since FL-cDNA sequences help to improve predicted gene structures, we constructed two FL-cDNA libraries and obtained c.a. 20,000 clones [10]. Our DB also includes public Iso-Seq and ESTs to capture various isoforms [11, 19–21]. Using RNA-seq technology, we aimed to reveal expression features of natural rubber biosynthetic genes. We previously obtained latex and non-latex (leaf, bark and petiole) in RRIM 600 genome cultivar and latex of other cultivars (RRIM 900 and PB350). Additionally, we downloaded latex and non-latex (bark and leaf) in RRIM 928 and latex under normal and stressed condition in RRII 105 cultivars. All data were re-analyzed with the same protocol [10] and descriptions of all transcriptomic data were summarized in additional file 1 (Table S1). All our original data can be downloaded from our web site.

**Table 1:** Data source and number of annotated genes of each database or experiment

Category	Contents	Number of annotated rubber genes	URL of the data source	Reference
Genome and proteins	Draft genome	84,443	http://matsui-lab.riken.jp/rubber/	[4]
Functional annotation	KEGG	61,453	http://www.genome.jp/	[15]
	NCBI Protein	64,585	https://www.ncbi.nlm.nih.gov/protein/	[16]
	Swiss-Prot	33,553	http://www.uniprot.org/uniprot/	[17]
	TrEMBLE	63,053		
	GO	35,247	http://www.geneontology.org/	[18]
Transcriptome	FL-cDNA	7704	http://matsui-lab.riken.jp/rubber/	[10]
	RNA-seq	42,614		[10] [23]
	CAGE	21,168		[4]
	ESTs	23,790^a^	http://www4a.biotec.or.th/rubber/ http://scarecrow.fmrp.usp.br/heveabr/	[19–21]
	Iso-Seq	17,668	http://www4a.biotec.or.th/rubber/	[11]

^a^Number of annotated rubber genes was calculated using three EST data sources and our original FL-cDNA

### Data search system

**Fig. 2:**
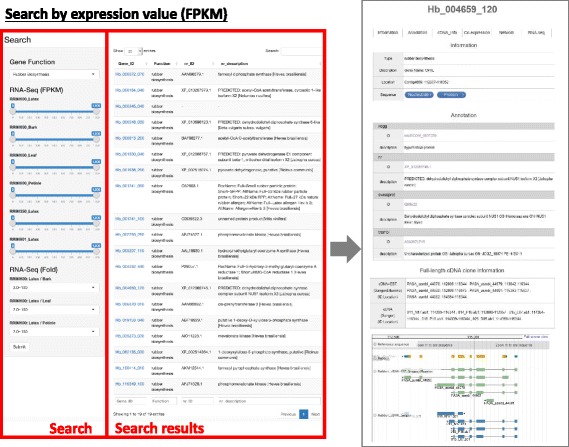
Screen capture and explanation of the expression search function. Users’ can easily select the expression threshold of each samples with FPKM value or Fold-change value

We provide three types of searching systems: keyword search, blast homology search and gene expression search. Since functional annotation of rubber genes is still limited, keyword search and homology search are not enough. We prepared gene expression search so that users can easily change gene expression threshold for samples and retrieve gene sets such as latex specific, for example. In our system, users can select gene expression value of FPKM (Fragments Per Kilobase Million) or select fold-change and obtain gene sets according users’ specific demands (Fig. 2).

### Genome browser

**Fig. 3:**
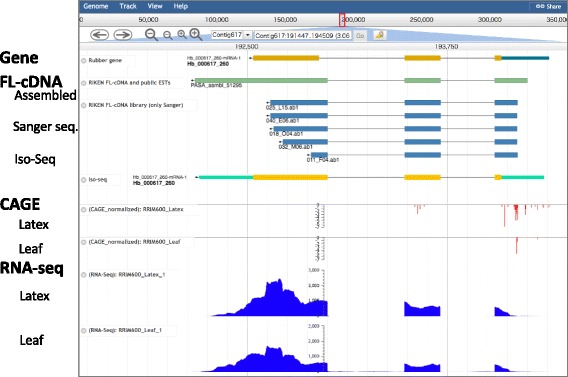
Gene, FL-cDNA, RNA-seq and CAGE data are visualized on the genome browser. We use the JBrowse fast and embeddable genome browser. Sequence information is also available from JBrowse

We visualized our multi-transcriptome data in a genome browser (Fig. 3). We made links for the genome browser on each gene web page. Users can visualize eleven RNA-seq, two FL-cDNA and three CAGE data and compare tissue specificity easily.

### Co-expressed genes and their network view

**Fig. 4:**
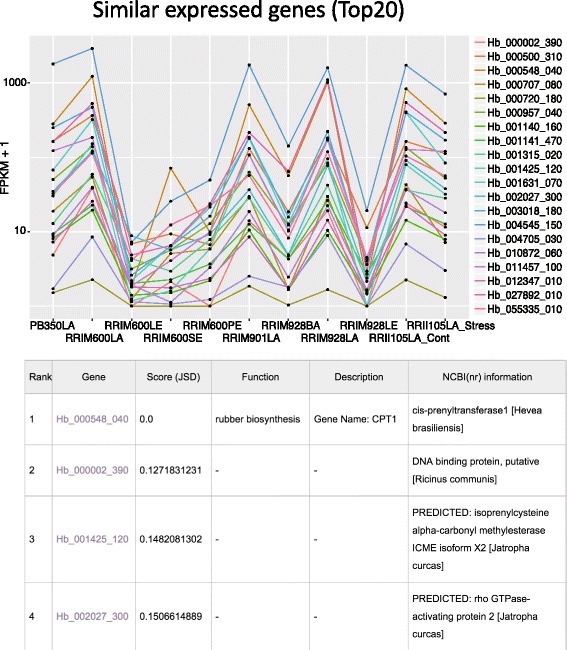
Top 20 of similar expressed genes of CPT1 (*cis*-prenyltransferase 1). The expression patterns (above) were calculated and visualized using R library cummeRbund. The bottom table shows functional annotation and scores (Jensen–Shannon divergence of similar expressed 10 genes

**Fig. 5:**
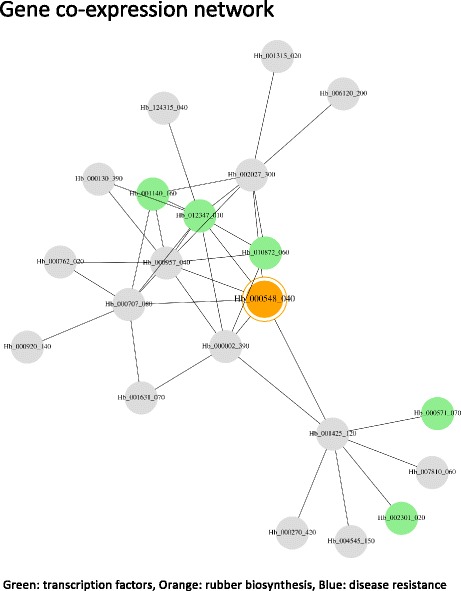
Two steps of co-expressed gene network of CPT1. Transcription factors are highlighted with green

Genes that are regulated by the same transcription factors or that work on the same biological pathways often show similar expression patterns. To suggest candidate genes that may have similar biological function with a user’s genes of interest, we calculated the top 20 similar expressed genes using our original and publically available 11 samples (RRIM600 cultivar: latex, bark, leaf and petiole, RRIM901 cultivar: latex, PB350 cultivar: latex [10], RRIM928 cultivar: latex, bark and leaf (SRP022257), RRIM105 latex: control latex and stressed latex (SRP017288)). To visualize the co-expressed gene network (Fig. 4), we used Gviz and highlighted transcription factors (Fig. 5).

### Rubber biosynthetic pathway search

**Fig. 6:**
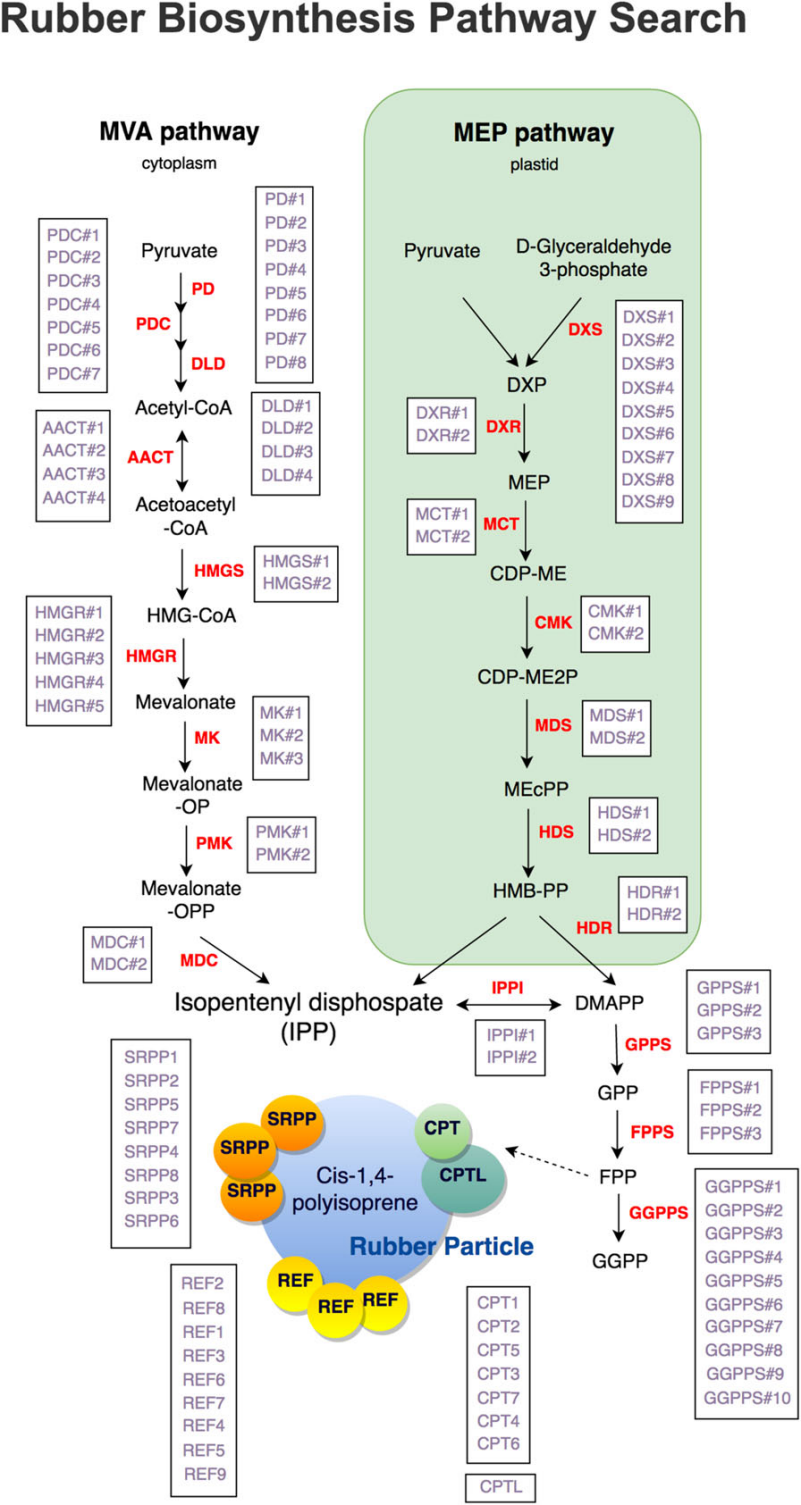
Rubber biosynthesis pathway search. Each gene is clickable and user can easily access to detailed gene page

The natural rubber biosynthetic pathway is not fully understood, especially regarding the polymerization process with isopentenyl diphosphates (IPPs). To clear known factors and their paralogous genes, we prepared a natural rubber biosynthetic pathway (Fig. 6). Users can easily understand the whole picture of the pathway and access gene information.

### Implementation of this web site

Rubber genome & transcriptome databased are currently running on Linux (Ubuntu 16.04.3 LTS) with the following environments: Perl (ver. 5.22.1), PHP (ver. 7.0.18), Python 2.7 and Apache HTTP server (ver. 2.4.18). As a relational database management system, we set up the MySQL (ver. 5.7.18). The co-expression network graph is drawn with Gviz (ver. 2.30.1-19.el7). Fast data access on the genome browser was implemented by JBrowse (ver. 1.12.3) [23]. Gene expression search was built with the Shiny package in R (https://shiny.rstudio.com/). We configured Shiny server to run multiple Shiny processes. Gene description pages are generated as static web pages.

## Discussion

Recently, RNA-seq is a powerful tool and the most widely spread transcriptome technology. However, RNA-seq is not suitable for predicting TSSs and full-length splice isoforms accurately. To obtain an entire picture of the rubber transcriptome, it is important to integrate multi-transcriptome data. In this DB, we integrated three types of transcriptome data: RNA-seq, FL-cDNA and CAGE. FL-cDNA provide us precise gene structures including findings of novel genes and novel alternative isoforms. To enrich the quality and quantity of splicing information it is necessary to know the correct protein sequence and estimate the protein function. Iso-Seq, PacBio single molecule long-read, is a powerful technology for this purpose. To know the genome-wide TSSs, we carried out CAGE and obtained precise TSSs in latex, leaf and bark. With CAGE data, we can observe tissue-dependent alternative TSSs, multiple TSSs in a gene and variations of TSS, such as strictly determined TSS in a base or others. In latex biosynthetic pathway, 1-deoxy-D-xylulose-5-phosphate synthase gene and pyruvate dehydrogenase gene showed the different TSS patterns between samples. Precise TSSs assist to find consensus sequences or motif sequences for transcription factors and their regulations.

As a next target, we plan to expand *cis*-elements that are predicted after co-expressed gene set.

## Conclusions

We have developed the rubber tree genome and transcriptome database, a comprehensive and searchable database of economically important plant, *H. brasiliensis*. To assist researchers and breeders in using the *H. brasiliensis* genome, we prepared a simple and user-friendly interface. We believe this database assists both industrial and academic researchers for rubber and important industrial close relatives such as *M. esculenta, R. communis and J. curcas*.

## Additional files

Additional file 1: Table S1. Detailed information on the transcriptome data. (PDF 12 kb)

## Abbreviations

CAGE: Cap analysis of gene expression
DB: Database
FL-cDNA: Full-length cDNA
Iso-Seq: Isoform sequencing
NGS: Next-generation sequencing
RNA-seq: RNA sequencing
TF: Transcription factor
TSS: Transcription start site
