# Supplementary material for: Construction of Pará rubber tree genome and multi-transcriptome database accelerates rubber researches
Source: BMC Genomics. 2018 Jan 19;19(Suppl 1):922. doi: 10.1186/s12864-017-4333-y (PMC5780850; doi:10.1186/s12864-017-4333-y)
Supplement: Supplementary file 1 — Detailed information on the transcriptome data. (PDF 12 kb) [file 12864_2017_4333_MOESM1_ESM.pdf]

Table S1. Detailed information on the transcriptome data

Category	Cultivar	Sampling		Num of raw reads	After assembly
FL-cDNA (library construction)	RRIM 600	latex, bark, leaf petiole	Sanger seq	28,726	19,487 clones
			Illumina	64,787,863	15,683 contigs
Iso-Seq	BPM24	leaf	PacBio	205,310	45,968 seq
EST	RRIM 600	male and female flowers, fruits, bark, latex, vegetative shoot apex		147,669	
RNA-seq	PB350	latex	Replicate 1	50,140,881	
			Replicate 2	54,181,272	
	RRIM 901	latex	Replicate 1	51,795,328	
			Replicate 2	50,904,032	
	RRIM 600	latex	Replicate 1	46,991,131	
			Replicate 2	52,074,445	
	RRIM 600	bark	Replicate 1	51,284,795	
			Replicate 2	46,559,165	
	RRIM 600	petiole	Replicate 1	46,246,170	
			Replicate 2	43,268,532	
	RRIM 600	leaf	Replicate 1	53,162,008	
			Replicate 2	47,181,675	Accessions
	RRIM 928	latex	Replicate 1	25,192,286	SRR854521
		bark	Replicate 1	84,943,813	SRR854520
		leaf	Replicate 1	24,789,161	SRR854522
	RRII105	latex control	Replicate 1	40,797,896	SRR620237
		latex stressed	Replicate 1	47,060,495	SRR620236

Highlighted samples are our original data. The others are public data.
